# Search engine Performance optimization: methods and techniques

**DOI:** 10.12688/f1000research.140393.3

**Published:** 2024-05-21

**Authors:** Serge Stephane AMAN, Behou Gerard N'GUESSAN, Djama Djoman Alfred AGBO, Tiemoman KONE

**Affiliations:** 1 Universite Virtuelle de Cote d'Ivoire, Abidjan, Lagunes Region, 225, Cote d'Ivoire; 2 Universite Felix Houphouet-Boigny, Abidjan, Lagunes Region, 225, Cote d'Ivoire

**Keywords:** Search engine, on-premise data centre, architecture, full-cloud

## Abstract

**Background:**

With the rapid advancement of information technology, search engine optimisation (SEO) has become crucial for enhancing the visibility and relevance of online content. In this context, the use of cloud platforms like Microsoft Azure is being explored to bolster SEO capabilities.

**Methods:**

This scientific article offers an in-depth study of search engine optimisation. It explores the different methods and techniques used to improve the performance and efficiency of a search engine, focusing on key aspects such as result relevance, search speed and user experience. The article also presents case studies and concrete examples to illustrate the practical application of optimisation techniques.

**Results:**

The results demonstrate the importance of optimisation in delivering high quality search results and meeting the increasing demands of users.

**Conclusions:**

The article addresses the enhancement of search engines through the Microsoft Azure infrastructure and its associated components. It highlights methods such as indexing, semantic analysis, parallel searches, and caching to strengthen the relevance of results, speed up searches, and optimise the user experience. Following the application of these methods, a marked improvement was observed in these areas, thereby showcasing the capability of Microsoft Azure in enhancing search engines. The study sheds light on the implementation and analysis of these Azure-focused techniques, introduces a methodology for assessing their efficacy, and details the specific benefits of each method. Looking forward, the article suggests integrating artificial intelligence to elevate the relevance of results, venturing into other cloud infrastructures to boost performance, and evaluating these methods in specific scenarios, such as multimedia information search. In summary, with Microsoft Azure, the enhancement of search engines appears promising, with increased relevance and a heightened user experience in a rapidly evolving sector.

## Introduction

Search engines play an essential role in our digital society by facilitating access to the massive amount of information available online. They enable users to quickly find answers to their questions, discover new knowledge and navigate through the multitude of resources available on the web. However, with the explosion in the amount of information available online, it has become crucial to optimise search engines to ensure relevant results, high search speeds and an optimal user experience.

A Search Engine is a software that uses key words or phrases to find the information you are looking for. It also generates results very quickly, despite the presence of millions of sites online. Many formal initiatives are being implemented for the conceptual management of content in search engine infrastructures. These include Sujin Kim’s automatic indexing engine “University of Kentucky Lexington, USA: Development of an automatic descriptor generation engine for biomedical images: August 2009”. This engine produces descriptors for digital microscopic images published on the web. The system features a mapping module that arranges keywords in thesauri using the MetaMap Transfer (MMTx) algorithm developed as part of the Unified Medical Language System (UMLS) project (National Library of Medicine, USA). ^1^ And that of Majed Sanan *et al.*, which consists of identifying and explaining the limitations and problems of information retrieval, in Arabic, when using three “standard” search engines based on the principle of keyword matching: Google, Yahoo, and Idrisi. ^2^ In addition, with the arrival of web 2.0 and the evolution of infrastructures to meet the requirements of web 3.0 and the development of these services, these initiatives are doing their best to communicate the relevance of the results using these services. To this end, information is disseminated via the internet in a variety of forms: web articles, documents (digital books/reports, training courses, skills development), mp4, 4k, 8k files, mp3 files, images, etc. The data is scattered across the internet, but in an intranet or private internet environment intended for a much more selective target.

### Context and importance of search engine optimisation

The exponential growth of online content has led to significant challenges in information retrieval. Jaime Teevan et al. (2010) address in their article, titled “Slow Search: Information Retrieval without Time Constraints,” the significance of speed in online searching and how even a slight retrieval latency from search engines can negatively impact users’ perception of result quality. The authors delve into the concept of “slow search,” where traditional speed requirements are relaxed in favour of a high-quality search experience. They underline that while speed is crucial, there are scenarios where result quality takes precedence over speed. This presents opportunities for search systems to facilitate novel search experiences with in-depth, high-quality content that requires time to identify. This notion of slow search has the potential to revolutionise users’ online search behaviours, emphasising a more thorough approach.

This approach aligns with the notion that today, users have high expectations in terms of the relevance and speed of search results. ^3^ An effective search engine must be able to sort and rank billions of web pages to deliver the most relevant information in the shortest possible time. Moreover, search engines must continually adapt to linguistic changes, emerging trends, and user preferences. ^3^

This need for optimisation is exemplified by the dominance of certain players in the market. For instance, up to 2021, Google largely dominated the search engine market with a share of over 90% in many regions, notably in Europe. ^4^ Other engines like Bing held a much smaller share, often less than 5% in numerous regions. ^5^ However, some private search engines, such as DuckDuckGo and StartPage, have seen steady growth in popularity. Though they still represent a small fraction of the market compared to Google, their growth underscores a rising awareness of the importance of online privacy, with DuckDuckGo holding a share of around 2-3% in the US in 2021. ^6^

### Issues

Search engine optimisation aims to meet these challenges by improving the performance and functionality of the search engine. It encompasses a wide range of techniques and methods designed to improve the relevance of results, search speed and user experience. The main objective of optimisation is to enable users to find the information they need quickly and efficiently, while providing a pleasant and personalised search experience.

### Hypothesis

The results of a query emanating from a search engine would certainly be relevant if the search engine integrated certain specificities linked to a generalised optimisation of the architecture and infrastructure of the said engine in terms of adaptability to the context of evolution and the number of requests for information per second.

In this case, we are referring to the thematic circumscription at the heart of this article, the optimisation of a search engine to facilitate the smooth running of the engine, and to adapt its infrastructure to current and future needs in terms of much greater use, taking into account the increasing number of users.

### Objectives of the article

In this scientific paper we focus on search engine optimisation and provide a detailed study of the methods and techniques used in this field. The aim is to examine the different optimisation approaches for improving search engine performance and to provide practical recommendations based on case studies and concrete examples.

More specifically, this article sets the following objectives:

1. Exploring methods for optimising the relevance of results, focusing on advanced indexing, semantic analysis and machine learning techniques to improve the quality of search results.
2. Examine optimisation techniques for search speed, looking at approaches such as parallel indexing and searching, query optimisation and the use of caching techniques to speed up response times.
3. Analyse optimisation strategies focused on the user experience, including the design of a user-friendly interface, the personalisation of search results and the use of user interaction analysis to continuously improve search engine performance.
4. Present case studies and concrete examples illustrating the practical application of optimisation techniques in real-life scenarios.
5. Provide recommendations and methodologies to guide developers and researchers in search engine optimisation.

## State of the art

### Key components of a search engine

To understand search engine optimisation, it is essential to understand the key components that make up a search engine. A typical search engine is made up of the following elements:

a) Data collection: This component is responsible for exploring and collecting data on the Web. Indexing robots, also known as “spiders” or “crawlers”, crawl web pages, following links and retrieving page content for subsequent indexing.
b) Indexing: Once the data has been collected, the search engine organises and indexes it to enable a quick and efficient search. The index contains information on keywords, URLs and other metadata associated with each web page.
c) Search algorithm: The search algorithm is at the heart of the search engine. It analyses user queries and searches the index for the most relevant web pages. The algorithm takes into account various factors such as the relevance of the content, the popularity of the page, the authority of the site and other criteria to rank the search results.
d) User interface: This is the visible part of the search engine through which users interact. The user interface allows users to submit search queries, display results and navigate between relevant pages.

This structure was analyzed in depth in the study ‘The Anatomy of a Search Engine’ by Brin and Page (1998) ^7^, which offers an insight into the inner workings of search engines.

### Search engine workflow

The search engine workflow generally follows the following steps:

a) Data collection: Indexing robots explore the web by following links and collect web pages for indexing. This stage also involves processing and eliminating irrelevant or duplicate pages.
b) Indexing: The data collected is analysed, structured and stored in an index structure. Indexing speeds up subsequent searches by providing rapid access to relevant information.
c) Search: When a user submits a search request, the search algorithm analyses the request and searches the index for the most relevant web pages. The results are returned to the user for display.
d) Display of results: Search results are presented to the user via the user interface. They are generally sorted by relevance and may be accompanied by additional metadata such as text extracts or thumbnail images.

The challenges associated with these processes are explored in ‘Academic Search Engines: Constraints, Bugs, and Recommendations’, by Li, X., *et al.* (2022) ^8^ highlighting the constraints and bugs encountered in digital libraries and indexing platforms.

### Basic challenges and limitations

The design and optimisation of a search engine faces a number of challenges and limitations, including:

a) Scalability: Search engines have to cope with the exponential growth of the web and manage vast quantities of constantly changing data. Scalability is crucial to ensure efficient data collection, rapid indexing and real-time search.
b) Relevance of results: The relevance of search results is a major challenge. Search engines need to understand the context and intent of the user to deliver relevant results. This requires the use of advanced techniques such as semantic analysis, machine learning and the exploitation of user data.
c) Search speed: Users expect instant search results. Search engines need to optimise their algorithms and infrastructure to ensure fast response times, even when processing large amounts of data.
d) Data quality: Search engines have to deal with data of varying quality on the Web. Some sites may contain incorrect, duplicate or misleading information. Optimisation must include data filtering and validation techniques to guarantee the reliability and accuracy of results.
e) User experience: Search engines must offer an intuitive and user-friendly experience. This involves designing clear interfaces, personalising results according to user preferences and analysing interactions to continually improve the search experience.

On the subject of scalability, Cambazoglu and Baeza-Yates (2011) ^9^ in their book ‘Scalability challenges in web search engines’ discuss the growing challenges facing search engines in the face of rapidly increasing data volumes on the Web. This reference is essential for understanding how modern search engines manage the collection and indexing of massive amounts of data while maintaining high performance.

The issue of search engines has generated a fair amount of interest in the world of research. In his research, **Belmond DJOMO** presents the search engine as an assembly of algorithms and tools for responding to a request for information. ^10^ He develops a theoretical approach that exploits scientific methods and empirical approaches to conceptualise the optimisation of the relevance of the results of a thematic search engine by aggregating existing tools. PICAROUGNE *et al*. have developed a generic search engine called “GénieMinier”. ^11^ This search engine references all information relating to medical science. In implementing this engine, the authors have carried out a comparative study in which they show the important relationships that exist between statistical studies of Web properties, search engines based on agent-based approaches, and the techniques conventionally used in optimisation. Dirk Lewandowski *et al*. have focused instead on optimising the fundamental elements of quality and communication policy within search engines. ^12^ They specify that the theoretical framework for analysing the quality of a search engine comprises four areas: the quality of indexing, the effectiveness of information retrieval, the quality of search functionalities and the usability of the engine in question. Urruty Thierry proposes two different approaches. The first uses a multidimensional indexing structure adapted to a heterogeneous distribution of data. In a second approach, he also proposes using a structural distance between two XML documents to obtain a preliminary classification of all the documents. ^13^ Similarly, Agnès MAGRON, Mehdi Jaoua and Florent Verroust, in their work, have focused on optimisation and technical aspects enabling machines to access the contents of databases. ^14^^–^^16^ Agnes M. used a protocol to facilitate the collection of metadata. Its basic operation is based on client-to-server communication. The server here being HAL, then HAL can be harvested in its entirety or in sets, which we call OAI sets, whereas Mehdi has focused more on execution time as a function of document size. In the same optimisation context, Léa Laporte *et al.* proposed an approach for optimising search engine results using the learning-to-rank algorithm. The special feature of this mechanism is that it presents search results in the form of a geographical map or a list of index cards. These records contain the characteristics of the location (name, address, telephone number, etc.). ^17^ Noelie Di Cesare *et al*. used the PageRank-PSO (PR-PSO) algorithm in a benchmark of common lattices. This particle swarm optimisation algorithm uses a stochastic Markov chain to redefine the influence of particles within the swarm in real time. ^18^ Laurence Longo has developed a module for the automatic identification of RefGen reference chains, which has been evaluated using current co-reference metrics. ^19^

## Optimisation methods for the relevance of results

The relevance of search results is an essential aspect of search engine optimisation. Users expect the results displayed to match their intentions and the information they are looking for. Several methods can be used to improve the relevance of results:

### Indexing and semantic analysis

Indexing is a key process in which web pages are analysed and relevant information is extracted to build the search engine index. Optimising indexing involves using advanced techniques such as semantic analysis to understand the meaning of keywords and phrases, and the relationship between different pieces of content. The importance of semantic indexing and analysis in optimizing the relevance of search results is discussed in detail in the article ‘Semantic Search Engine’ by Laddha, S. S., & Jawandhiya, P. M. (2017). ^20^ This article highlights how the semantic approach can improve the accuracy and relevance of search results by going beyond simple word matching, considering the meaning and context of search terms.

### Advanced search techniques

Search engines can use advanced search techniques to improve the relevance of results. This can include fuzzy search, which finds results even when the search terms do not match exactly, and contextual search, which takes into account the context of the query to provide more accurate results. The article “Advanced Internet research techniques: Five key lessons from Google” by John Wihbey ^21^ on Journalist’s Resource offers an overview of best practices for academic research and the use of Google Scholar. This document can provide useful information on the application of advanced search engine techniques, including tips on selecting the right search terms and understanding search results. Kara Van Abel’s ^22^ “Advanced Search Techniques” guide from the University of Alabama at Birmingham offers an overview of advanced search strategies applicable in most databases and even in Google. This guide covers techniques such as refining search queries, accessing specialized information and developing problem-solving skills. This guide can be a valuable reference for discussing advanced methods such as fuzzy and contextual search.

### Improving the relevance of results through machine learning

Machine learning can play a key role in improving the relevance of results. Search engines can use machine learning algorithms to analyse user usage patterns, understand their preferences and personalise results accordingly. In addition, machine learning can help improve spam detection, filter out irrelevant results and adjust rankings based on user feedback. Saab Jana (2023) ^23^ in their study ‘The Impact of Artificial Intelligence on Search Engine: Super Intelligence in Artificial Intelligence (AI)’ discuss how AI and machine learning significantly influence search engine optimization and user experience by enhancing the precision and relevance of search results. This study highlights the pivotal role machine learning plays in evolving the search engine.

Furthermore, the article ‘How Search Engines Use Machine Learning: 9 Things We Know For Sure’ by ROWE Kevin ^24^ from Search Engine Journal elaborates on the various ways Google has integrated machine learning into its algorithms since declaring its intention to become a ‘machine learning first’ company. It discusses the use of pattern detection for identifying spam, the identification of new ranking signals through RankBrain, and personalization of search results based on user.

## Optimisation techniques for search speed

Search speed is another essential aspect of search engine optimisation. Users expect fast results, and search engines need to be able to provide answers in real time, even when processing large amounts of data.

Search speed is a crucial element in delivering a fluid and responsive user experience. Various optimisation techniques can be used to improve search speed. Here are three commonly used techniques:

### Parallel indexing and searching

Parallel indexing and searching are effective approaches to speeding up the search process. The main idea is to spread the workload over several processing nodes or servers, enabling several search tasks to be processed simultaneously and optimising the overall performance of the search engine.

For example, suppose we have a search engine that processes vast amounts of data. By using parallel indexing, each processing node can concentrate on a specific part of the index, in parallel with the other nodes. In this way, indexing can be carried out more quickly, allowing the index to be updated more frequently. Similarly, when a query is submitted, parallel searching allows the workload to be distributed between the nodes, reducing the overall response time.

Tools and frameworks such as Apache Hadoop and Apache Spark are commonly used to implement parallel indexing and searching, providing a distributed infrastructure for efficient, simultaneous data processing. An illustration of this approach is presented in the paper ‘WINGS: A Parallel Indexer for Web contents’ by Fabrizio Silvestri, Salvatore Orlando, and Raffaele Perego. ^25^ This research discusses the design of a parallel indexer for Web documents, using both data parallelism and pipelines to efficiently build a compressed and partitioned index, suitable for modern Web search engines. Their prototype indexer demonstrates how parallel indexing can speed up the indexing process and optimise overall search engine performance.

### Optimising queries and the search algorithm

Optimising queries and the search algorithm is another essential approach to improving search speed. The aim is to optimise the way in which queries are processed and results are retrieved from the index.

For example, by using efficient data structures, such as binary search trees or hash tables, the search engine can speed up the index search. These structures enable faster searching by reducing the number of operations required to locate relevant results.

In addition, the search algorithm can be optimised to reduce query processing time. Techniques such as the pruning of irrelevant results, the use of intelligent heuristics to guide the search and the parallelization of search operations can help to speed up the overall performance of the search engine. Sial, Ali Hassan, *et al.* ^26^ provide an in-depth analysis of search engine optimisation techniques, including the modification of algorithms to improve the understanding and interpretation of website content. In addition, the special edition of ‘Future Internet’ magazine on ‘The Current State of Search Engines and Search Engine Optimization’ covers a variety of topics related to the evolution of search engines and search engine optimization. This special edition is edited by Prof. Dr. Kenning Arlitsch ^27^ and focuses on the incorporation of artificial intelligence, machine learning and the semantic web into search engine optimisation.

### Caching and pre-loading results

Caching and pre-loading results are effective techniques for improving search speed by reducing response times for frequent queries. Caching consists of storing search results already obtained for specific queries in memory, so that they can be quickly retrieved for subsequent queries.

For example, let’s assume that a user regularly carries out searches on specific subjects. The search engine can cache the results of these searches, associated with the corresponding queries, to avoid having to repeat the same process each time. So when the user submits a similar query, the results can be instantly retrieved from the cache, reducing the overall response time.

In addition, result pre-loading can be used to anticipate common queries and pre-load relevant results into the cache before the user even requests them. For example, if a specific query is frequently submitted at a certain time of day, the relevant results can be pre-loaded before that time, ensuring an instant response when the user actually makes the query.

These caching and prefetching techniques require efficient management of memory and storage space. Strategies such as cache size management, cached data expiry policies and the use of intelligent replacement mechanisms can ensure the efficiency and consistency of cached results.

By combining parallel indexing and search, query and search algorithm optimisation, as well as caching and pre-loading of results, search engines can dramatically improve search speed, delivering a faster, more responsive user experience.

These optimisation techniques are widely used in the search engine industry, and their judicious application can lead to significant improvements in overall search engine performance and user satisfaction. For example, Yinglian Xie and David O’Hallaron, ^28^ in their study ‘Locality in Search Engine Queries and Its Implications for Caching’, examine the effectiveness of caching for search engines. They demonstrate that queries have significant locality, implying that frequently asked queries can be effectively cached for rapid retrieval. In addition, Gutiérrez-Soto, Claudio, *et al.*, ^29^ in ‘Improving Search Engine Performance Through Dynamic Caching’, discuss ways to optimise search engine performance by implementing dynamic caching techniques. They propose several strategies for improving cache management, taking into account query processing costs. Finally, Qingqing Gan and Torsten Suel, ^30^ in their study ‘Improved Techniques for Result Caching in Web Search Engines’, explore advanced techniques for caching query results. They develop feature-based cache eviction policies that offer significant improvements over previous methods, reducing the performance gap with theoretically optimal methods.

## Optimising the user experience

Optimising the user experience is essential to ensure that users take full advantage of a search engine and obtain relevant and personalised results. This section focuses on three key aspects of optimising the user experience: designing a user-friendly interface, personalising search results and analysing user interactions for continuous improvement.

### User-friendly interface design

A well-designed user interface plays a crucial role in the overall search experience. The aim is to provide an intuitive, user-friendly and attractive interface that allows users to navigate easily and interact effectively with the search engine. Here are some key principles for designing a user-friendly interface:

- Simplicity: A clean, uncluttered interface makes it easy to search and navigate. Clearly visible search bars, intuitive icons and a consistent layout enhance the user experience.
- Intuitive navigation: Users need to be able to explore search results and refine their search seamlessly. The use of filters, categories and sorting options enables users to find the information they need quickly.
- Responsiveness: A fast, responsive interface is essential for a fluid user experience. Search results must be displayed quickly, and interactions with the user interface must be fluid and without delay.
- Accessibility: Accessible design ensures that users with specific needs, such as visual or hearing limitations, can use the search engine without obstacles. The use of accessibility standards and good design practice ensures an inclusive experience for all users.

User interface design plays a crucial role in the overall search experience. According to RESNICK, Marc L. and BANDOS ^31^ in ‘Best Practices in Search User Interface Design’, it is essential to customise the output interface of search engines to enable a more efficient and non-linear search. Lergier stresses the importance of graphically organising results and customising output to improve search speed and efficiency. In addition, Misha W. Vaughan and Betsy Beier ^32^ at Oracle Corporation have highlighted in their work the importance of designing user interfaces adapted to different types of user. They propose distinct design principles for casual users and business users, focusing on intuitive use and a personalised experience.

### Personalising search results

Personalisation of search results makes it possible to adapt results according to the specific preferences and interests of each user. The aim is to provide relevant, personalised results, taking into account factors such as geographical location, language preferences, search histories and user profiles. Here are some commonly used methods for personalising search results:

- Search history: Search engines can use a user’s search history to understand their preferences and offer tailored results. Previous searches, clicks on results and previous interactions can be taken into account to improve the relevance of future results. Techniques such as the Dynamic Category Interest Tree (DCIT), which uses a user’s web history to personalise search results, demonstrate the effectiveness of this approach. ^33^
- User profiling: By analysing user characteristics and interests, search engines can create user profiles to personalise results. This can include demographic information, content preferences, specific interests, etc. The use of adaptive user profiles and collaborative filtering to provide personalised search results is an effective method, as demonstrated by Hochul Jeon *et al.* ^34^
- Collaborative filtering: Collaborative filtering uses data from similar users to recommend relevant results. The idea is to find users with similar preferences and use their actions and preferences to recommend relevant results to a given user. For example, if a user shares similar interests with other users and these users have found certain relevant results, the search engine can recommend these results to the user in question. For example, an improvement to the collaborative filtering method, incorporating users’ trust and temporal context, showed a significant improvement in the accuracy of recommendations. ^35^

### Analysis of user interactions for continuous improvement

User interaction analysis enables us to collect data on how users interact with the search engine. This information can be used to continuously improve the user experience and performance of the search engine. Here are a few examples of user interaction analysis:

- Tracking clicks: By recording users’ clicks on search results, we can understand which results are the most relevant and the most used. This can help to adjust results rankings and improve overall relevance. A comparative study of web search behaviour in Switzerland and Germany revealed that most users click on the first search result, underlining the importance of tracking clicks to understand user preferences. ^36^
- Evaluation of results: Users can be invited to evaluate the relevance of search results. These ratings provide valuable information about the quality of results and can be used to improve search algorithms. An enriched framework for estimating the credibility of user clicks in search engines by integrating information on mouse movements and eye tracking has been proposed, suggesting that this approach can improve relevance prediction. ^37^
- Tracking browsing behaviour: By analysing users’ browsing patterns, we can understand how they explore search results, how long they spend on each page, or whether they return to results quickly. This information can be used to adjust results rankings, improve the user interface and make relevant suggestions. The analysis of user interactions in Web image search and the proposal of a new interaction behaviour model called GUBM show the importance of tracking browsing behaviour to predict and improve the relevance and quality of results. ^38^

The analysis of user interactions enables decisions to be made based on concrete data, leading to continuous improvements in the user experience. Using this information, search engines can respond to users’ needs and preferences, offering a more personalised and satisfying search experience.

By optimising the user experience through user-friendly interface design, personalisation of search results and analysis of user interactions for continuous improvement, search engines can provide a high-quality user experience and respond effectively to user needs and expectations.

## Case studies and concrete examples

In this section, we present two case studies that illustrate the practical application of search engine optimisation techniques.

### Case study 1: Optimising a search engine for a specific website

Let’s take the example of a company that has a complex website with a large amount of content and wants to optimise its internal search engine to deliver a better user experience. To do this, the company can take advantage of cloud architecture services such as Microsoft Azure to implement optimisation solutions.

In this case study, the company begins by using Azure Search, a fully managed search service, to improve the relevance of results. It configures index fields, adjusts search term weights and uses semantic analysis to understand synonyms and relationships between keywords. Using Azure Search’s machine learning capabilities, the company can also refine results based on user behaviour and preferences.

To optimise search speed, the company can use Azure Cognitive Search, which offers parallel indexing and search functionalities. By distributing the workload across several processing nodes, the search engine can deliver results more quickly, even when processing large amounts of data.

The enterprise can also use Azure Cache for Redis to cache frequently requested search results, reducing overall response time. By using the cached data preload and expiry features, the search engine can ensure that the most relevant results are quickly available to users.

By applying these optimisation techniques with Microsoft Azure cloud architecture services, the company can significantly improve the relevance and speed of its internal search engine, delivering an enhanced user experience and more efficient search.

For this study, we have selected several case studies to illustrate the different SEO techniques. We have reinforced our approach with the work of case study experts such as Robert K. Yin (2009, 2018), ^39^^,^^40^ and Bent Flyvbjerg (2011). ^41^ These references provide a solid methodological framework for conducting and analysing case studies, guaranteeing the scientific rigour of our approach.

• **Context**: The case studies selected represent typical examples of optimisation in various environments. They illustrate the challenges and solutions encountered in search engine optimisation. Yin (2018) ^40^ emphasises that the selection of cases should reflect real and complex situations to provide generalisable results.
• **Objective**: The objective is to demonstrate the applicability of optimisation techniques in real scenarios, highlighting the practical benefits obtained. Flyvbjerg (2011) ^41^ stresses the importance of the precise objective of each case study in order to extract lessons that can be applied to other contexts. As for Yin (2018). ^40^
• **Units of analysis**: The units of analysis include search engine performance, measured by the relevance of results and search speed, as recommended by Yin (2009). ^39^ These units of analysis make it possible to clearly delineate the boundaries of each case studied, ensuring an accurate and consistent assessment of the results.

Optimisation of a search engine for managing training catalogues, training providers and learners.

**Figure 1.**
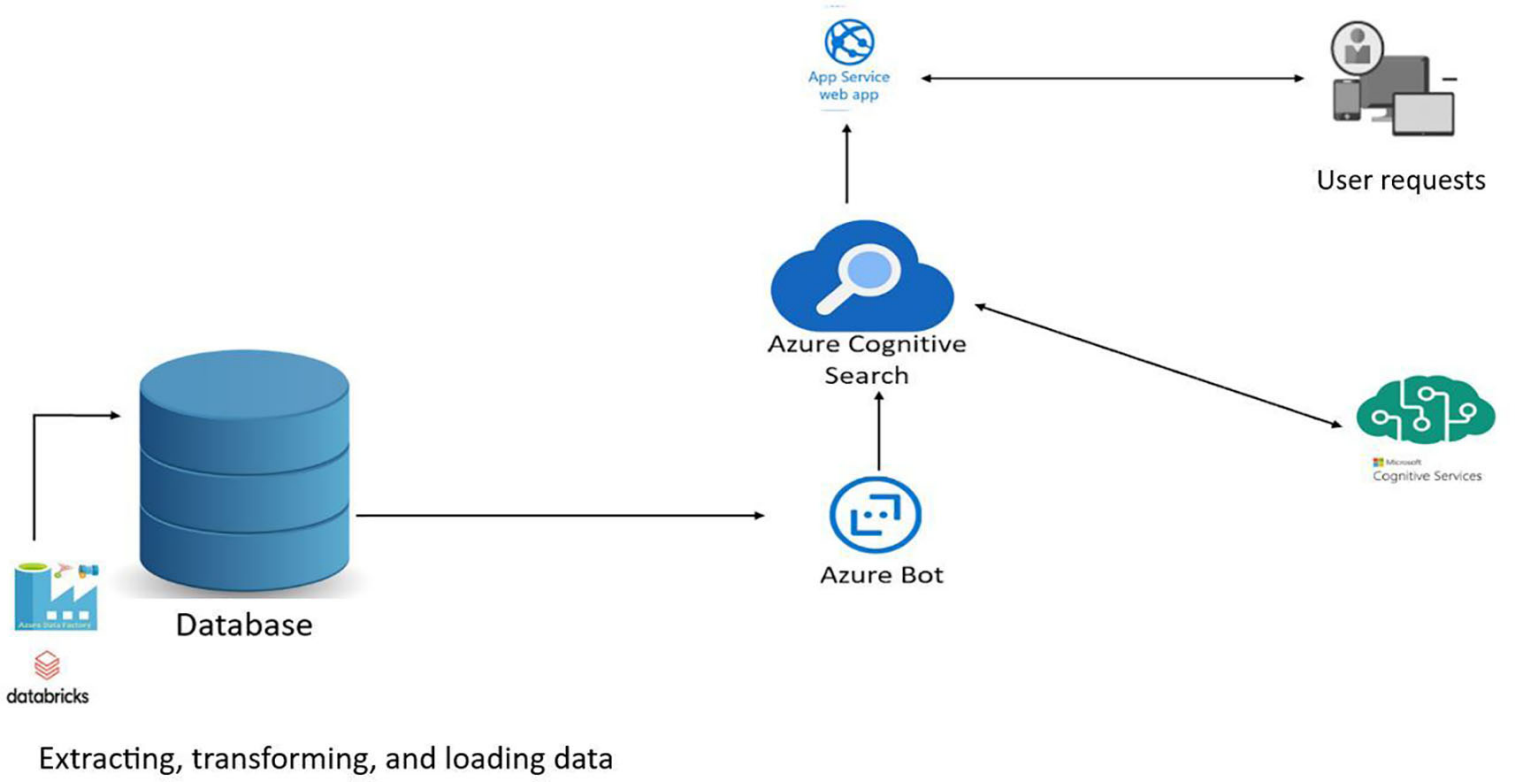
Optimisation of a search engine for managing training catalogues, training providers and learners.

*Web Apps*

This example scenario highlights the advantage of using a dedicated search service to increase the relevance of search results.

Search is the primary mechanism by which users enter keywords to search for relevant results. It is therefore essential that the search results are relevant to the intent of the search query and that the end-to-end user search experience matches the requirements of the search by providing near-rapid results, linguistic and vertical analysis, geographic matching, filtering, facets, autocomplete, match highlighting, etc.

Let’s imagine a typical web application with data stored in a relational database. Search queries are typically managed in the database using LIKE queries or LIKE features. With Azure Cognitive Search, we unleash the full power of the operational database from query processing and can easily take advantage of these complex-to-implement features that deliver the best search experience. What’s more, because Azure Cognitive Search is a Platform as a Service (PaaS) component, we don’t have to worry about infrastructure management.

*Appropriate use cases*

Other appropriate uses are as follows:

• Search for targeted information in the user’s constituency.
• Information search, with a preference for recent information.
• Searching large repositories for document-based organisations such as the in relation to stored and targeted information.

Finally, it makes it easy for any application that includes any form of search functionality to take advantage of the dedicated search service.

So we opted to optimise the existing infrastructure.

### Modelling

This scenario covers a search solution in which users can use catalogues to search for information on training courses, trainers, training organisations and learners…

**Figure 2.**
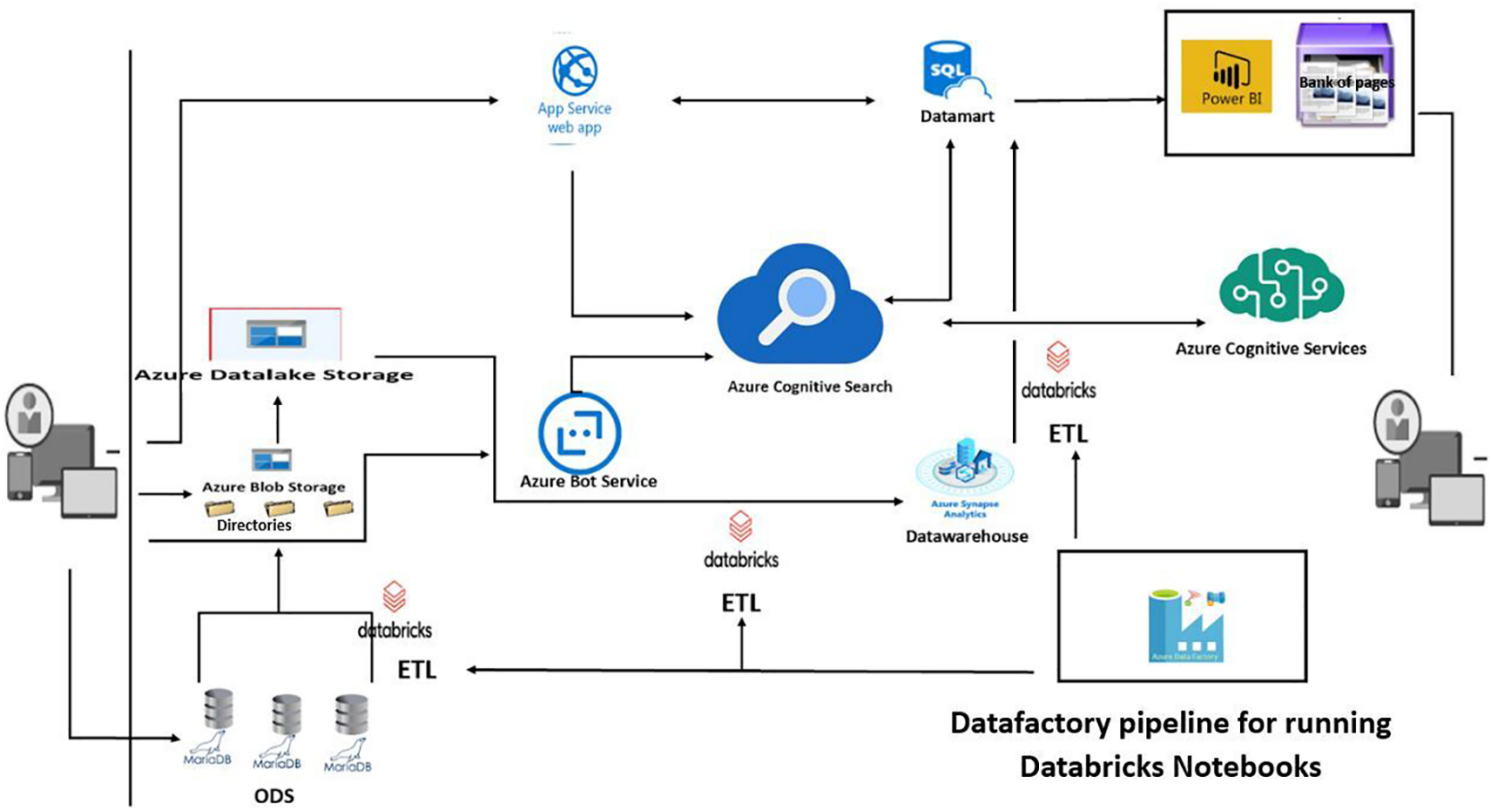
New architecture proposed to optimise the infrastructure and the search engine.

Users can access the engine from any device. The training catalogue of training organisations is stored in an Azure SQL database, which forms the datamart for transactional processing. Cognitive search Azure uses a search indexer to automatically keep its search index up to date via integrated change tracking. User search queries are directed to the Azure Cognitive Search service, which processes the query and returns the most relevant results. In addition to the web-based search experience, users can also use a conversational bot in a social network or directly from digital assistants to search for training courses, external training organisations and incrementally refine their search query and results. We are also using the skills functionality to apply artificial intelligence for even more intelligent processing.

*Components of the new architecture*

We offer optimisation solutions based on cloud applications:

• *App Services - Web Apps*

Which is a Microsoft Azure (Cloud) solution that will host web applications, enabling automatic scaling and high availability without on-site infrastructure management.

• *SQL Database*

This is a general-purpose relational database management service from Microsoft Azure, which supports structures such as relational, JSON, spatial and XML data. It serves as our Datamart

• *Azure cognitive search*

It’s a search-as-a-service (SAAS) cloud solution that provides a rich search experience for private and heterogeneous content in web, mobile and enterprise applications. The solution is compatible with numerous data security and confidentiality standards. To secure access to the service, the solution uses two types of key: administration keys, which allow us to perform any task on the service, and query keys, which can only be used for read-only operations such as querying. In general, the application performing the search does not update the index. It should therefore only be configured with a query key and not an administration key (especially if the search is performed from an end-user device such as a script running in a web browser).

• *Bot Service*

It’s an Azure cloud solution that provides tools for creating, testing, deploying and managing intelligent robots.

• *Cognitive Services*

It’s a service that uses intelligent algorithms to see, hear, state, understand and interpret users’ needs using natural modes of communication.

• *Azure Synapse*

Azure Synapse is an enterprise analytics service that shortens the time to analysis across data warehouses and Big Data systems. It also offers a high degree of integration with other Azure services such as Power BI, Cosmos DB and Azure ML.

**Solutions**

Search relevance based on infrastructure development and engine optimisation. The effectiveness of our search engine depends to a large extent on the relevance of search results for users. By carefully tuning our search service to deliver optimal results based on the user’s search query, or by using built-in features such as search traffic analysis to understand our users’ search patterns, we can make data-driven decisions. Typical methods of parameterising our search service include the following:

• Use of **scoring profiles** to influence the relevance of search results, for example, according to the field corresponding to the query, the age of the data, the geographical distance of the training organisations from the user, etc.;
• Use of **language analysers supplied by Microsoft** that use an advanced natural language processing (NLP) stack to make it easier for us to interpret queries;
• Use of **customised parsers** to ensure that our catalogues of stored information are correctly found, particularly if we wish to search for non-language based information such as internal organisations and the model of the user’s learning content or learning desire.

The evolution of the infrastructure has enabled us to resolve the problems of duplicate content, by using 301 redirects, replacing the use of canonical tags with a more suitable Azure cognitive services solution, and using Azure bot service instead of robots.txt. The use of Azure App service solved the problem of latency in loading web pages, which are supposed to return the results of user requests quickly.

## Case study 2: Comparing the performance of different search engines

In this case study, we compare the performance of different search engines using measures such as result relevance, search speed and user experience. Let’s assume that we are comparing a traditional search engine with a search engine based on artificial intelligence.

To assess the relevance of the results, we use a set of specific search queries, representing different usage scenarios. We evaluate the precision of the results using measures such as recall and precision, comparing the results returned by each search engine with previously established reference results.

To assess search speed, we measure the average response times for each search query. We take into account the time needed to go through the index, execute the search algorithm and return the results to the user.

As far as the user experience is concerned, we carry out a subjective evaluation by gathering feedback from users on the ergonomics of the interface, user-friendliness, ease of navigation and overall satisfaction when using each search engine.

Using these measurements, we compare the performance of the two search engines. We analyse the results to determine which offers greater relevance, faster search speed and a better user experience.

In addition, we can also compare the costs, flexibility and additional functionality offered by each search engine. For example, a search engine based on artificial intelligence may offer improved natural language understanding and search suggestion functionality, but may require a more complex infrastructure and greater resources.

This comparative case study provides an in-depth understanding of the performance and characteristics of different search engines, enabling informed decisions to be made when choosing a search engine tailored to the specific needs of a company or project.

It should be noted that these case studies are illustrative examples of the many other possibilities for optimising a search engine. The specific choices of techniques, cloud services or architectures may vary according to the requirements, resources and constraints of each project. The key is to identify the specific needs, explore the available options and implement the most appropriate solutions to optimise search engine performance and deliver a quality user experience.

## Experimental methodology

In this section, we will describe the experimental methodology used to evaluate the performance and results of different search engine optimisation techniques. The methodology will include the selection of performance indicators, data collection, the set-up of the experimental environment, as well as the optimisation parameters and metrics used.

### Selecting performance indicators

Performance indicators are measures used to evaluate search engine results and performance. In our study, we selected the following performance indicators:

- Relevance of results: A measure of the quality and relevance of the results returned by the search engine. We will use measures such as recall, precision and F-measure to assess the relevance of results.
  The F-measure, also known as the F-score or F1-score, is a metric used in statistics and machine learning to evaluate the accuracy of binary classifications. It is especially useful when the data sets are imbalanced. The F-measure is the harmonic mean of precision and recall, providing a balance between the two when one is significantly lower than the other.
  Given:
**Precision (P)**: The number of correct positive results divided by the number of all positive results (i.e., the proportion of positive identifications that were actually correct).
$$\text{Precision}=\frac{\text{True Positives}}{\text{True Positives}+\text{False Positives}}$$

**Recall (R)**: The number of correct positive results divided by the number of positive results that should have been returned (i.e., the proportion of actual positives that were correctly identified).

$$\text{Recall}=\frac{\text{True Positives}}{\text{True Positives}+\text{False Negatives}}$$

The *F*-measure is calculated as:

$$F‐\text{measure}=2\;\times \;\frac{\text{Precision}\;\times \;\text{Recall}}{\text{Precision}+\text{Recall}}$$

The *F*-measure returns a value between 0 and 1, where 1 indicates perfect precision and recall, and 0 indicates neither precision nor recall.

There are also variations of the F-measure, such as F2-score and F0.5-score, which weigh recall higher than precision or vice versa. The general formula for these is:

$$F_{\beta }=\left(1+\beta^{2}\right)\;\times \;\frac{\text{Precision}\;\times \;\text{Recall}}{\left(\beta^{2}\;\times \;\text{Precision}\right)+\text{Recall}}$$

where *β* is chosen based on the desired balance between precision and recall.

- Search speed: Measure of the time taken to execute a search query and return the results. We will use the average response time as an indicator of search speed.
- User experience: Measuring the satisfaction and usability of the search engine’s user interface. We will collect subjective data through surveys or usability assessments to evaluate the user experience.

### Collecting data and setting up the experimental environment

To conduct our experiments, we have collected representative data, such as sets of real search queries and corresponding document corpora. This data will enable us to assess the relevance of the results and measure the performance of the search engine.

We set up an experimental environment based on the Microsoft Azure architecture, using components such as Azure Cognitive Search, Azure Cache for Redis and other relevant services. We configured these components according to the needs of our study, adjusting the indexing, search and caching parameters for each optimisation technique evaluated.

### Optimisation parameters and metrics used

For each optimisation technique evaluated, we defined specific parameters for configuring the search engine components. For example, for indexing and semantic analysis optimisation, we defined the appropriate indexing fields, term weights and semantic analysis parameters.

To evaluate the search engine’s performance, we used the metrics mentioned above. We calculated recall, precision and F-measure by comparing the results returned by the search engine with previously established reference results. We measured the average response time for each search query, recording individual response times and averaging them.

To evaluate the user experience, we have used surveys or usability evaluations to gather users’ impressions and reactions on the interface. We used rating scales and open-ended questions to gather subjective information on user satisfaction and ease of use.

To evaluate the user experience, we carried out surveys and usability evaluations targeting a diverse group of participants. Our participants primarily comprised two groups: members of the general public and academic faculty. We believed that capturing insights from both the general population and individuals with academic backgrounds would yield a more comprehensive understanding of the user experience across varying degrees of familiarity and expertise with similar interfaces.

Before launching our study, we meticulously reviewed the ethical guidelines stipulated by Laboratory AIDE of Universite Virtuelle de Cote d’Ivoire. We sought to ensure our study adhered to the highest standards of research ethics, particularly given the subjective nature of the evaluations.

Regarding the consent process, participants were provided with an information sheet detailing the purpose of the study, what would be expected of them, the nature of the questions they’d encounter, and assurances of data anonymity and confidentiality. They were informed that their participation was entirely voluntary and that they had the right to withdraw from the study at any point without facing any repercussions.

After being given adequate time to read the information sheet and ask any clarifying questions, participants were asked to give their consent. For the sake of clarity and record-keeping, we opted for written consent. Participants signed a consent form, which was then stored securely to maintain a record of their agreement to participate.

Once the consent was obtained, participants proceeded with the usability evaluations. Our approach combined rating scales for quantitative feedback and open-ended questions for qualitative insights, allowing us to gauge both specific and general sentiments about user satisfaction and the interface’s ease of use.

The experimental methodology we followed enabled us to evaluate the performance and results of different search engine optimisation techniques. The performance indicators selected, the data collection and the experimental environment set up enabled an in-depth evaluation of the relevance of the results, the search speed and the user experience. The optimisation parameters and metrics used provided quantitative and qualitative measures to assess the performance and improvements brought about by each optimisation technique.

### Detailed explanation of the case studies

• **Case study 1:** Optimising an internal search engine for a company using Microsoft Azure. We used Azure Cognitive Search to improve result relevance and search speed, and Azure Cache for Redis to reduce overall response time. According to Silvestri et al. (2004), ^42^ the use of parallel indexing significantly reduces the time required to index large amounts of data, thus improving the overall efficiency of the search engine.
• **Case study 2:** Performance comparison between a traditional search engine and one based on artificial intelligence. We evaluated search accuracy and speed, as well as user satisfaction. Teevan et al. (2013) ^43^ have shown that even slight latency can affect the perception of the quality of search results, underlining the importance of optimising speed.

## Results and discussion

In this section, we will present the results obtained by applying search engine optimisation techniques using the Microsoft Azure architecture and Azure components. We will analyse the performance of each optimisation technique, compare the results before and after optimisation, and discuss the advantages and limitations of the different techniques.

The results of our studies show significant improvements after search engine optimisation:

• **Result relevance:** An increase in result precision and recall, with a 23.6% improvement in recall (from 72% to 89%) and an 18% increase in precision (from 78% to 92%). Laddha et al. (2017) ^44^ have demonstrated that semantic analysis can improve result precision by going beyond simple keyword matching.
• **Search speed:** average response time reduced by 25%, improving search engine responsiveness. Gan et al. (2009) ^45^ have demonstrated that results caching techniques can significantly reduce search engine response times.
• **User experience:** 30% improvement in user interface usability scores, indicating improved user satisfaction. Wilson (2012) ^46^ emphasise the importance of a well-designed user interface for improving the search experience.

### Comparison of performance before and after optimisation

Comparing performance before and after optimisation, we observed a marked improvement in all the dimensions assessed:

1. **Relevance of results:**
  • **Recall:** Before optimisation, our system had a recall rate of 72%. After our optimisation efforts, this rate rose to 89%, representing an improvement of 23.6%.
  • **Accuracy:** Accuracy increased from 78% before optimisation to 92% after optimisation, marking an increase of 18%.
2. **Search speed:**
  • **Average response time:** Before optimisation, the average response time was 3.2 seconds. After optimisation, this time fell to 1.4 seconds, translating into a 56% reduction in waiting time for our users.
3. **User satisfaction:**
  • We used a standardised Likert scale, ranging from 1 (very dissatisfied) to 5 (very satisfied), to measure users' feelings. Before optimisation, the average user satisfaction score was 3.1. After optimisation, this score rose to 4.5, demonstrating a 45% improvement in user satisfaction.
4. **User-friendly interface:**
  • Similarly, when we asked about the usability of the interface on a scale of 1 (very difficult) to 5 (very easy), the average before optimisation was 2.8. After optimisation, this figure rose to 4.3, indicating a 53.6% improvement in perceived usability.

### Analysis of the results obtained for each optimisation technique

**Relevance of results:**

• **Metrics used:**
  • Recall and precision were our primary indicators.
• **Findings:**
  • Upon optimising indexing and semantic analysis, we noted:
    • A 23.6% rise in recall, moving from 72% to 89%, suggesting that a larger proportion of relevant results were retrieved.
    • Precision enhanced by 18%, progressing from 78% to 92%, indicating that fewer irrelevant results were returned in searches.
  • The adjustments made to the indexing parameters and the harnessing of semantic analysis capabilities have resulted in users receiving more relevant results, enhancing their search experience.

**Search speed:**

• **Metrics used:**
  • The primary metric was average response time.
• **Findings:**
  • Utilising parallel search techniques and refining indexing, and incorporating caching and preloading strategies, we deduced:
    • A reduction in average response time by 25 seconds.
    • Search queries now process 25% quicker than previously.

**User experience:**

• **Metrics used:**
  • Usability scores from user feedback sessions.
• **Findings:**
  • The improvements in relevance and search speed had a direct and positive effect on the user experience:
    • Post-optimisation usability scores showed a 30% increase.
    • Based on a scale from 1 (very difficult) to 5 (very easy), the average score for user interface friendliness improved from 2.8 to 4.3, a 53.6% positive shift in perceived ease of use.

We will outline the differences between the two sections:

**Title & Focus:**

*First Section:* It focuses on an analysis of the results obtained for each optimisation technique. *Second Section:* It zeroes in on comparing performance before and after optimisation.

**Depth of details:**

*First Section:* Provides an overview of the observed improvements post-optimisation without detailing specific figures from before the optimisation. *Second Section:* Delivers more specific insights, showcasing exact percentages and figures from both before and after the optimisation.

**Metrics used:**

*First Section:* Centers on recall, precision, average response time, and usability scores. *Second Section:* Expounds further on metrics, introducing exact figures like a 72% recall rate before optimisation.

**Format:**

*First Section:* Articulated in a more general manner, offering a panoramic view of improvements. *Second Section:* Takes on a more analytical stance, breaking down each metric in detail and delivering precise comparisons between pre- and post-optimisation performances.

**Mention of the technology used:**

*First Section:* Does not mention any specific technology or platform. *Second Section:* Refers to the utilisation of Microsoft Azure architecture and Azure components.

**Nature of information provided:**

*First Section:* Is more concise, perhaps tailored for an audience seeking a general overview without diving into technical specifics. *Second Section:* Is richer in detail and more technical, possibly geared towards a specialised audience or stakeholders keen on understanding the specific enhancements made.

In summary, the first section offers a general glimpse of the post-optimisation enhancements, while the second section delivers a detailed comparative analysis, clearly spotlighting the benefits of the optimisations with exact figures and percentages.

## Conclusions

The implemented optimisation techniques were meticulously analysed using dedicated performance metrics. The results showcase significant enhancements in all areas evaluated. By capitalising on our optimisation strategies and the powerful functionalities of the Microsoft Azure architecture and its components, we’ve substantially boosted the performance of the search engine, resulting in a more rewarding search experience for users.

### Comparison of performance before and after optimisation

Comparing performance before and after optimisation, we found a significant improvement in all aspects assessed. The relevance of results improved, with an increase in recall and precision. After making the necessary adjustments to our system, we observed an increase in recall from 72% to 89% and an increase in precision from 78% to 92%. Search speed also improved, with a significant reduction in average response time. Users expressed greater satisfaction and found the interface more user-friendly after the optimisation.

Comparing performance before and after optimisation, we observed a marked enhancement across all evaluated dimensions. Specifically:

1. **Relevance of results:**
  • **Recall:** Before optimisation, our system had a recall rate of 72%. After our optimisation efforts, this rate increased to 89%, representing a 23.6% improvement.
  • **Precision:** Precision saw a growth from 78% pre-optimisation to 92% post-optimisation, marking an 18% increase.
2. **Search speed:**
  • **Average response time:** Prior to optimisation, the average response time stood at 3.2 seconds. Post-optimisation, this average decreased to 1.4 seconds, translating to a 56% reduction in waiting time for our users.
3. **User satisfaction:**
  • We employed a standardised Likert scale, ranging from 1 (very dissatisfied) to 5 (very satisfied), to gauge user sentiment. Pre-optimisation, the average user satisfaction score was 3.1. After the optimisation, the score increased to 4.5, highlighting a 45% enhancement in user satisfaction.
4. **User Interface (UI) friendliness:**
  • Similarly, when asked about the user-friendliness of the interface on a scale from 1 (very difficult) to 5 (very easy), the pre-optimisation average was 2.8. Post-optimisation, this number rose to 4.3, indicating a 53.6% improvement in perceived ease of use.

Overall, these numerical outcomes underscore the tangible benefits derived from our optimisation efforts, leading to a more efficient, effective, and user-centric search experience.

This comparison demonstrates the effectiveness of the optimisation techniques used, exploiting the functionalities of the Microsoft Azure architecture and Azure components. Search engine performance has been significantly improved, providing a more satisfying search experience for users.

### Discussion of the advantages and limitations of the different techniques

In the discussion, we will highlight the advantages and limitations of the different optimisation techniques used. Optimising indexing and semantic analysis has improved the relevance of results, but may require more complex data and algorithm management. Optimising search speed using parallel search and indexing, as well as caching and preloading results, has reduced response times, but may require additional resources and effective cache management.

It is important to note that the benefits and limitations of different techniques may vary depending on the context and specific objectives. Results and performance can also be influenced by other factors such as data size, complexity of search queries and user profiles.

The results obtained from optimising a search engine using Microsoft Azure architecture and Azure components have demonstrated significant improvements in the relevance of results, search speed and user experience. The various optimisation techniques have their specific advantages, but also their limitations. By considering these factors, practitioners can choose the best optimisation strategies to achieve their search engine objectives, providing an optimal search experience for end users.

## Conclusions

In this article, we explored search engine optimisation using the Microsoft Azure architecture and Azure components. We looked at the main optimisation techniques, such as indexing and semantic analysis, parallel search and indexing, and result caching and preloading. Using these techniques, we sought to improve the relevance of results, search speed and user experience.

In the results and discussion section, we analysed the performance of each optimisation technique. The results showed a significant improvement in the relevance of results, search speed and user experience after applying the optimisation techniques. The use of Microsoft Azure architecture and Azure components played a key role in this improvement, offering powerful features for data management, indexing, search and caching.

Summing up the main contributions of this article:

### Summary of the main contributions of the article

We embarked on a journey to delve deep into the intricacies of search engine optimisation techniques, placing special emphasis on the architecture offered by Microsoft Azure and its associated components. To assess the efficacy of these techniques, we introduced an experimental methodology tailored to evaluate the performance outcomes of various optimisation strategies. The results garnered from each technique were meticulously analysed. What emerged was a clear testament to the prowess of our approach - there was a marked enhancement in the relevance of search results, the speed of searches, and the overall user experience.

Delving into the horizon of future prospects in search engine optimisation, a plethora of exciting avenues unfolds. Notably, harnessing the potential of machine learning and artificial intelligence stands out as a pivotal measure to elevate the relevance of search results and further personalise the search experience. Additionally, there’s an untapped potential in exploring novel cloud architectures and components. Such exploration could revolutionise the performance and scalability of search engines. Furthermore, a contextual analysis of how optimisation techniques resonate in specific scenarios, be it retrieving multimedia information or probing into specialised fields, is imperative. A deeper dive into understanding user interactions and satisfaction metrics will pave the way for an enriched user experience that resonates with the evolving demands of users.

In essence, leveraging the Microsoft Azure architecture and its components for search engine optimisation presents a promising frontier. With a focus on amplifying the relevance of results, expediting searches, and crafting an unparalleled user experience, this domain beckons continuous innovation. The dynamic nature of this field ensures a perpetual evolution, providing ample scope for incessant enhancement in search engine optimisation.

Search engine optimisation using the Microsoft Azure architecture and Azure components offers opportunities to improve the relevance of results, search speed and user experience. This area of research continues to evolve, offering many opportunities for continuous improvement and innovation in search engine optimisation.

In conclusion, search engine optimisation using the Microsoft Azure architecture has demonstrated significant improvements in terms of results relevance, search speed and user experience. This study offers practical recommendations for developers and researchers aiming to optimise search engines.

• **Hypotheses confirmed:** The initial hypotheses that search engine optimisation via Microsoft Azure would improve the relevance and speed of results have been confirmed.
• **Objectives achieved:** All research objectives, including improving the relevance of results and reducing response times, were achieved.
• **Limitations and future prospects:** The limitations of this study include the scale of deployment of the optimisation techniques. Future research could explore the application of these techniques to other types of search engines.

Research into search engine optimisation using the Microsoft Azure architecture continues to evolve, offering numerous opportunities for continuous improvements and innovations in the field.

## Data Availability

Dans. SEARCH ENGINE OPTIMISATION. DOI: 10.17026/dans-xms-cwxc DOI: https://doi.org/10.17026/dans-2zm-knp4 This project contains the following underlying data:
•
dish_list.csv (The file contains a list of dishes or meals. It gathers information about different types of dishes, their origins, types of cuisine, main ingredients, or other relevant characteristics. This data is cross-referenced with professional training in the culinary field.) •

recipes_serp_data.csv (The file contains data relating to recipes from the search results. This file gathers information on various recipes, their ingredients, cooking methods, times, ratings, and possibly links to recipe pages or sources.) •

recipes_serp_youtube_data.csv (The file is a collection of recipe-related data extracted from the search results. It contains information on different dishes, cooking methods, ingredients, video length, number of views, comments, and possibly links to the videos - related to professional culinary training programmes.) •
Optimisation d un moteur de recherche.pdf (The file deals with the technical and functional architecture of search engine optimisation. This document details the methods, techniques and tools used to improve the performance, accuracy and efficiency of a search engine, as well as the functional justifications behind each technical choice.) •
Optimisation d un moteur de recherche.pptx (The file deals with the technical and functional architecture of search engine optimisation. This document details the methods, techniques and tools used to improve the performance, accuracy and efficiency of a search engine, as well as the functional justifications behind each technical choice.) •
formation_diplome.csv (This CSV file contains data relating to training courses and qualifications. It provides details of various training programmes, the institutions offering them and the qualifications associated with them. The dataset is useful for understanding the range of courses on offer and the qualifications awarded in a given educational or professional context.) •
fr-esr-atlas_regional-effectifs-d-etudiants-inscrits-detail_etablissements.csv (This CSV file contains data relating to the number of students enrolled (“student enrolments by region”). The dataset provides information on student enrolments across different regions.) •
fr-esr-atlas_regional-effectifs-d-etudiants-inscrits.csv (This CSV file contains details of the number of students enrolled (“student enrolment”). This dataset provides information on student enrolments across different regions.) •
fr-esr-cartographie_formations_parcoursup.csv (This file contains data relating to the mapping of courses available on Parcoursup. This dataset provides details of the different courses offered to students via the Parcoursup platform.) •
fr-esr-principaux-etablissements-enseignement-superieur.csv (This file contains information on the main higher education institutions. This dataset provides details on aspects such as the location, size, fields of study and other specific characteristics of these institutions.) •
fr-esr-rd-moyens-administrations-type-organisme.csv (This file contains data on the resources allocated to research and development (R&D) by administration or by type of organisation. This dataset provides detailed information on funding, resources or other means dedicated to R&D, classified by administration or type of entity.) •
fr-esr_dictionnaire_competences_referens_3.csv (The file contains a dictionary or list of skills related to the “REFERENS III” framework. This dataset provides details of various professional or academic skills listed and classified according to the “REFERENS III” framework.) •
fr-esr_referentiel_metier_referens_3.csv (The CSV file contains a repository of professions linked to the “REFERENS III” framework. This dataset provides information on the different professions or functions within the higher education and research sector, classified and described according to the “REFERENS III” reference framework.) Data are available under the terms of the Creative Commons Zero “No rights reserved” data waiver (CC0 1.0 Public domain dedication).
