# Supplementary material for: Search engine Performance optimization: methods and techniques
Source: F1000Res. 2024 May 21;12:1317. Originally published 2023 Oct 12. [Version 3] doi: 10.12688/f1000research.140393.3 (PMC11157186; doi:10.12688/f1000research.140393.3)
Supplement: Peer review history

# Reviewer report: Reviewer response for version 3

**DOI:** 10.5256/f1000research.166647.r280946

**Published:** 2024-06-06

**Authors:** Carlos Lopezosa

**Affiliations:** 1 University of Barcelona, Barcelona, Spain

Dear editors,

 I consider that the authors have applied the expected changes to their paper with effort, so I believe this research research can now be indexed.

 Best regards,

 Carlos.

Is the work clearly and accurately presented and does it cite the current literature?

Yes

If applicable, is the statistical analysis and its interpretation appropriate?

I cannot comment. A qualified statistician is required.

Are all the source data underlying the results available to ensure full reproducibility?

Yes

Is the study design appropriate and is the work technically sound?

Partly

Are the conclusions drawn adequately supported by the results?

Yes

Are sufficient details of methods and analysis provided to allow replication by others?

Yes

Reviewer Expertise:

Search engine optimization, web visibility, artificial intelligence

I confirm that I have read this submission and believe that I have an appropriate level of expertise to confirm that it is of an acceptable scientific standard.

# Reviewer report: Reviewer response for version 3

**DOI:** 10.5256/f1000research.166647.r280947

**Published:** 2024-05-27

**Authors:** Mike Nkongolo Wa Nkongolo

**Affiliations:** 1 University of Pretoria, Gauteng, South Africa

Results are not well presented (missing figures and tables). Experimental data must be visualized.

 There is also missing comparison with existing studies.

 The authors state the following: " To conduct our experiments, we have collected representative data, such as sets of real search queries and corresponding document corpora.". But this data is not presented or visualize in the manuscript.

 The Graphical User Interface of the search engine must be presented.

 How many participants are involved in the study? what is their demographic information? this has not been specified.

Is the work clearly and accurately presented and does it cite the current literature?

Partly

If applicable, is the statistical analysis and its interpretation appropriate?

Partly

Are all the source data underlying the results available to ensure full reproducibility?

Partly

Is the study design appropriate and is the work technically sound?

Partly

Are the conclusions drawn adequately supported by the results?

Partly

Are sufficient details of methods and analysis provided to allow replication by others?

No

Reviewer Expertise:

SEO

I confirm that I have read this submission and believe that I have an appropriate level of expertise to state that I do not consider it to be of an acceptable scientific standard, for reasons outlined above.

## References

[1] : News Classification and Categorization with Smart Function Sentiment Analysis. *International Journal of Intelligent Systems* .2023;2023: 10.1155/2023/1784394 1-24 10.1155/2023/1784394

# Reviewer report: Reviewer response for version 2

**DOI:** 10.5256/f1000research.161303.r258260

**Published:** 2024-05-15

**Authors:** Carlos Lopezosa

**Affiliations:** 1 University of Barcelona, Barcelona, Spain

We are facing very interesting research that addresses the improvement of search engines through Microsoft Azure infrastructure and its associated components. To achieve this, it aims to examine various optimization approaches to enhance search engine performance and provide practical recommendations based on case studies and concrete examples.

 Overall, the state of the art is correct, providing not only an overview of the topic but also aiding in understanding the work within its context. In this regard, the bibliography is comprehensive.

 Throughout the methodological section, the selection of the sample is adequately explained. However, there are recommendations for improvement regarding the selection of the case studies:

 1.    The explanation and description of the different case study is correct but can be improved. To do this, we propose supporting your selection with a bibliographical reference from an expert in case studies (some of the most important, in case it may be useful to the authors, are Robert K. Yin, Eisenbarth, among others). Authors may consider these or other authors, whichever they deem most appropriate.

 2.    Throughout the methodological section, the sample selection is adequately explained. To provide even stronger support and to prevent any potential questioning of the case selection, we recommend responding systematically and uniformly to the following:

 a. Background: Provide information or data that helps establish the significance of the selection. 

 b. Purpose of this selection: Explain the specific purpose behind this choice. 

 c. Units of analysis that will define the case boundaries: Offer greater precision regarding the units of analysis that will help delineate the case boundaries.

 The research results are very interesting, relevant, and useful. In addition, the graphs are very timely and help to better understand the results.  

 The discussion section, although interesting, can be improved. It should be a space to debate the results obtained with other related studies, and in this case, it's not fulfilled; that's why there's a recommendation to try to improve this section by addressing these considerations.

 Finally, while the conclusions effectively summarize the main findings, for transparency, it is recommended to review the hypotheses and research objectives at this point to evaluate their degree of fulfillment. Explicitly reaffirming them in the conclusions would improve clarity for the reader. It is also recommended that the conclusions allude to possible limitations and future research.

Is the work clearly and accurately presented and does it cite the current literature?

Yes

If applicable, is the statistical analysis and its interpretation appropriate?

I cannot comment. A qualified statistician is required.

Are all the source data underlying the results available to ensure full reproducibility?

Yes

Is the study design appropriate and is the work technically sound?

Partly

Are the conclusions drawn adequately supported by the results?

Yes

Are sufficient details of methods and analysis provided to allow replication by others?

Yes

Reviewer Expertise:

Search engine optimization, web visibility, artificial intelligence

I confirm that I have read this submission and believe that I have an appropriate level of expertise to confirm that it is of an acceptable scientific standard, however I have significant reservations, as outlined above.

## References

[1] : Case study research and applications. *SAGE Publications,Inc* .2018;

[2] : Case study research: Design and methods. *SAGE Publications,Inc* .2009;

[3] : Case Study Research: Design and Methods. *London: Sage Publications,Inc* .2014;

[4] : “Case Study”. In: Denzin, N.; Lincoln, Y. Strategies of Qualitative Inquiry. *pp. 169-204./London: Sage,* .2013;

[5] : How to do your Case Study. *London: Sage,* .2011;

# Reviewer report: Reviewer response for version 2

**DOI:** 10.5256/f1000research.161303.r238304

**Published:** 2024-02-13

**Authors:** Dimitrios Giomelakis

**Affiliations:** 1 University of Cyprus, Nicosia, Cyprus

The author(s) have addressed the points raised in my previous review, and I am satisfied that they have met the requested revisions. The manuscript has been significantly improved after the revision.

Is the work clearly and accurately presented and does it cite the current literature?

Partly

If applicable, is the statistical analysis and its interpretation appropriate?

Yes

Are all the source data underlying the results available to ensure full reproducibility?

Yes

Is the study design appropriate and is the work technically sound?

Yes

Are the conclusions drawn adequately supported by the results?

Yes

Are sufficient details of methods and analysis provided to allow replication by others?

Yes

Reviewer Expertise:

online journalism, news, SEO, web analytics, media technologies

I confirm that I have read this submission and believe that I have an appropriate level of expertise to confirm that it is of an acceptable scientific standard.

# Reviewer report: Reviewer response for version 1

**DOI:** 10.5256/f1000research.153738.r229214

**Published:** 2024-05-16

**Authors:** Mike Nkongolo Wa Nkongolo

**Affiliations:** 1 University of Pretoria, Gauteng, South Africa

Search engine optimization: methods and techniques is the proposed title.

 "The focus of this paper is on search engine optimization and to provide a detailed study of the methods and techniques used in this landscape."

 Add 4 keywords.

 Referencing requires improvement and consistency in this paper.

 Support your claim with referencing. 

 Include research questions and hypothesis in the introduction.

 State the research contribution clearly in the introduction.

  Detail the research methodology. Was PRISMA used? experimental methodology? How many papers selected for the systematic literature review (inclusion and exclusion criteria?)?

  Discuss figures in detail and refer to them in the text.

 Structure the articles into numbered sections and subsections.

 Expand the literature review with additional articles such as: 

 Nkongolo Wa Nkongolo M et al.(2023 ^1^)

 Barik K et al.(2023 ^2^)

 Liu G et al.(2023 ^3^)

 Compare the obtained results with the existing studies (tabulate your results)

 Tabulate the obtained results and produce various plots to be discussed.

 Equation should be labelled with numbers (e.g., (1), (1.2), (2), etc.)

 The application of Machine Learning and Natural Language Processing techniques for search engine optimization should be discussed in detail.

 Azure components required explanation.

Is the work clearly and accurately presented and does it cite the current literature?

Partly

If applicable, is the statistical analysis and its interpretation appropriate?

Partly

Are all the source data underlying the results available to ensure full reproducibility?

Partly

Is the study design appropriate and is the work technically sound?

Partly

Are the conclusions drawn adequately supported by the results?

Partly

Are sufficient details of methods and analysis provided to allow replication by others?

No

Reviewer Expertise:

Search Engine optimization.

I confirm that I have read this submission and believe that I have an appropriate level of expertise to state that I do not consider it to be of an acceptable scientific standard, for reasons outlined above.

## References

[1] : News Classification and Categorization with Smart Function Sentiment Analysis. *International Journal of Intelligent Systems* .2023;2023: 10.1155/2023/1784394 1-24 10.1155/2023/1784394

[2] : LSTM-DGWO-Based Sentiment Analysis Framework for Analyzing Online Customer Reviews. *Comput Intell Neurosci* .2023;2023: 10.1155/2023/6348831 6348831 10.1155/2023/6348831 36820054 PMC9938778

[3] : An Automatic Privacy-Aware Framework for Text Data in Online Social Network Based on a Multi-Deep Learning Model. *International Journal of Intelligent Systems* .2023;2023: 10.1155/2023/1727285 1-23 10.1155/2023/1727285

# Reviewer report: Reviewer response for version 1

**DOI:** 10.5256/f1000research.153738.r229254

**Published:** 2024-01-02

**Authors:** Dimitrios Giomelakis

**Affiliations:** 1 University of Cyprus, Nicosia, Cyprus

I found the paper interesting, but I would suggest two main improvements that could enhance its quality.

 1) In the 'State of the Art' section and throughout discussions on elements such as the key components of a search engine/search engine workflow, optimization methods for the relevance of results, techniques for search speed optimization, and strategies for enhancing the user experience, it is imperative for the authors to include relevant references in each of these sections. This step is essential to enhance and bolster the academic quality of this paper.

 2) Upon reading the title 'Search Engine Optimization,' I initially expected the paper to delve into the process of improving the quality and quantity of website traffic (organic results, on-page/off-page SEO). However, the paper primarily focuses on enhancing the performance and functionality of a search engine. To avoid any potential confusion, the authors should explicitly mention and clarify this distinction.

 Unfortunately, I am not familiar with Microsoft Azure, including its architecture and various components. As a result, I cannot review the second (and more practical) part of this paper.

Is the work clearly and accurately presented and does it cite the current literature?

Partly

If applicable, is the statistical analysis and its interpretation appropriate?

Yes

Are all the source data underlying the results available to ensure full reproducibility?

Yes

Is the study design appropriate and is the work technically sound?

Yes

Are the conclusions drawn adequately supported by the results?

Yes

Are sufficient details of methods and analysis provided to allow replication by others?

Yes

Reviewer Expertise:

online journalism, news, SEO, web analytics, media technologies

I confirm that I have read this submission and believe that I have an appropriate level of expertise to confirm that it is of an acceptable scientific standard, however I have significant reservations, as outlined above.
